# Quantifying *p*CO_2_ in biological ocean acidification experiments: A comparison of four methods

**DOI:** 10.1371/journal.pone.0185469

**Published:** 2017-09-28

**Authors:** Sue-Ann Watson, Katharina E. Fabricius, Philip L. Munday

**Affiliations:** 1 Australian Research Council Centre of Excellence for Coral Reef Studies, James Cook University, Townsville, Queensland, Australia; 2 Australian Institute of Marine Science, Townsville, Queensland, Australia; Helmholtz-Zentrum fur Ozeanforschung Kiel, GERMANY

## Abstract

Quantifying the amount of carbon dioxide (CO_2_) in seawater is an essential component of ocean acidification research; however, equipment for measuring CO_2_ directly can be costly and involve complex, bulky apparatus. Consequently, other parameters of the carbonate system, such as pH and total alkalinity (A_T_), are often measured and used to calculate the partial pressure of CO_2_ (*p*CO_2_) in seawater, especially in biological CO_2_-manipulation studies, including large ecological experiments and those conducted at field sites. Here we compare four methods of *p*CO_2_ determination that have been used in biological ocean acidification experiments: 1) Versatile INstrument for the Determination of Total inorganic carbon and titration Alkalinity (VINDTA) measurement of dissolved inorganic carbon (C_T_) and A_T_, 2) spectrophotometric measurement of pH_T_ and A_T_, 3) electrode measurement of pH_NBS_ and A_T_, and 4) the direct measurement of CO_2_ using a portable CO_2_ equilibrator with a non-dispersive infrared (NDIR) gas analyser. In this study, we found these four methods can produce very similar *p*CO_2_ estimates, and the three methods often suited to field-based application (spectrophotometric pH_T_, electrode pH_NBS_ and CO_2_ equilibrator) produced estimated measurement uncertainties of 3.5–4.6% for *p*CO_2_. Importantly, we are not advocating the replacement of established methods to measure seawater carbonate chemistry, particularly for high-accuracy quantification of carbonate parameters in seawater such as open ocean chemistry, for real-time measures of ocean change, nor for the measurement of small changes in seawater *p*CO_2_. However, for biological CO_2_-manipulation experiments measuring differences of over 100 μatm *p*CO_2_ among treatments, we find the four methods described here can produce similar results with careful use.

## Introduction

Since the beginning of the Industrial Revolution, the oceans have absorbed about a third of all anthropogenic carbon dioxide (CO_2_) emissions released into the atmosphere [1, 2]. In seawater, CO_2_ reacts to form carbonic acid (H_2_CO_3_) which dissociates into hydrogen (H^+^) and bicarbonate ions (HCO_3_^-^). This process, known as ocean acidification, results in increased concentrations of CO_2(aq)_, H^+^ and HCO_3_^-^, and reductions in carbonate ion (CO_3_^2-^) concentration and the saturation state of seawater with respect to calcite and aragonite. As a result of ocean acidification, surface oceans are now approximately 0.1 pH units lower and 30% more acidic than 250 years ago [3]. Ocean chemistry is changing faster than any time during the last 65 million years [4], and possibly the last 300 million years [5]. Under current CO_2_ emissions rates (Representative Concentration Pathways, RCP 8.5 scenario), atmospheric CO_2_ levels are projected to exceed 900 ppm by the end of this century [6] and seawater pH projected to decline a further 0.14–0.43 units [3].

In the surface ocean, *p*CO_2_ is rising at the same rate as atmospheric CO_2_ [7]. Recent models suggest seasonal *p*CO_2_ cycles will be amplified as atmospheric CO_2_ levels rise, which means that *p*CO_2_ in the surface ocean may be considerably higher than in the atmosphere for many months each year and open ocean regions could exceed 1000 μatm *p*CO_2_ before the end of the century [8]. Coastal waters exhibit particularly large seasonal and diel variation in pH and *p*CO_2_ (e.g. [9, 10]), and consequently, anthropogenic amplification of the *p*CO_2_ cycle in coastal waters is likely to be even more pronounced [11].

Seawater *p*CO_2_ can be assessed: 1) by direct measurement of CO_2_ in a gas volume equilibrated with seawater using gas analysers equipped with non-dispersive infrared (NDIR) sensors, or 2) indirectly by measuring two parameters of the seawater carbonate chemistry system and then calculating *p*CO_2_. Direct NDIR measurement of CO_2_ is often conducted using equilibrators that are specifically designed for the continuous measurement of CO_2_, such as on ships (e.g. [12]), or modified to measure CO_2_ in a small volume of air in a closed loop that is equilibrated with CO_2_ in water. Commonly, seawater *p*CO_2_ is calculated using any pair of carbonate chemistry parameters. Frequently used parameters include pH, total alkalinity (A_T_), and dissolved inorganic carbon (C_T_).

Measurements of seawater carbonate chemistry parameters vary in 1) measurement time, 2) accuracy, 3) cost, and 4) the time lag to obtain results (e.g. zero if results are obtained immediately, or potentially months later in the case of water sample batch processing; Table 1). For example, pH is commonly measured immediately *in situ* or *in vitro* using a relatively low-cost pH meter and electrode, or spectrophotometrically *in vitro* after addition of a pH indicator dye. A_T_ and C_T_ are measured *in vitro*, usually from mercuric chloride poisoned water samples, and generally require more complex, customised, bulky and costly laboratory equipment such as an automatic titrator or Versatile INstrument for the Determination of Total inorganic carbon and titration Alkalinity (VINDTA), respectively; although it is possible to perform titrations manually with a lower-cost pH meter and electrode (or pH indicator), and burette. Systems such as VINDTA are complicated but very precise, while other methods that are easier to use may not reach the same accuracy.

**Table 1:** Summary table of methods used in this study. Sample measurement time refers to measurements made during this study, with the upper end of the time range allowing for machine warm-up.

Method	Time lag to obtain results	Approx. sample measurement time	Approx. cost of equipment (USD in 2016)
VINDTA C_T_	Often long as samples are usually batch processed, often after the experiment	10–60 min	$75,000
Spectrophotometric pH_T_	Shortly after real-time	10–45 min	$3,000–10,000
Electrode pH_NBS_	Real-time	1–3 min	$1,000
NDIR CO_2_ equilibrator	Shortly after real-time (corresponding to equilibration time)	30–60 min	$4,000

Choosing appropriate measurement techniques to achieve the required precision and accuracy for carbonate chemistry parameters should be the primary consideration; however, the number and frequency of measurements required for a study is also an important consideration in measurement technique choice, and will relate to the number of treatment levels and replicates within the experimental design. For biological ocean acidification experiments, measurement accuracy will vary depending on the research question addressed and target *p*CO_2_ treatment levels employed, and accuracies of ±50 μatm *p*CO_2_ are likely commonly suitable for biological CO_2_-manipulation experiments measuring differences of more than 100 μatm *p*CO_2_ among treatments. Furthermore, the measurement of some carbonate chemistry parameters requires sophisticated equipment, which is not always accessible, particularly at remote field sites. Many biological ocean acidification studies are now conducted in more remote locations such as field research stations, and submarine CO_2_ vents and seeps (e.g. [13–16]), and include large, highly-replicated ecological studies. In such field based environments where access to specialist chemical oceanography equipment is limited, researchers need to repeatedly monitor seawater carbonate conditions during their experiments, often with multiple treatments and upwards of 50 or more replicates running simultaneously that require monitoring on a daily or more frequent basis. Field researchers therefore need techniques that can provide reliable, cost-effective, real-time estimates of *p*CO_2_ to maintain their experiments.

Here we assess a range of methods commonly available to determine seawater carbonate chemistry in biological ocean acidification experiments. We consider four parameters that are commonly measured to constrain the CO_2_ system in seawater: A_T_, C_T_, pH, and *p*CO_2_ [17], and we compare four different methods to determine the *p*CO_2_ of seawater: 1) C_T_ and A_T_, 2) spectrophotometric pH_T_ and A_T_, 3) electrode pH_NBS_ and A_T_, and 4) a portable CO_2_ equilibrator with a NDIR gas analyser to measure CO_2_ directly *in situ*. We assess measurement time, accuracy, costs and the time lag to obtain results for the four methods. We focus particularly on *p*CO_2_ determination firstly, because quantifying *p*CO_2_ is particularly important in designing biological manipulation experiments relevant to emissions trajectories such as the Intergovernmental Panel on Climate Change’s Representative Carbon Pathways (IPCC RCPs) and secondly, because *p*CO_2_ is very sensitive to small changes in other carbonate parameters making it a useful measure for this comparative approach. We also expand on the CO_2_ equilibrator technique by describing a simple method for the direct, *in situ* measurement of CO_2_ in seawater using a portable CO_2_ equilibrator coupled to a NDIR gas analyser.

## Materials and methods

### Experimental system and seawater manipulation

This study was conducted at Lizard Island, Great Barrier Reef, Australia (S 14° 41’, E 145° 28’) at the Australian Museum’s Lizard Island Research Station flow-through aquarium facility. Water from the ocean was pumped into an environmentally-controlled room where seawater flowed into a 60 L header tank fitted with a powerhead to circulate the water. Seawater from the header tank was gravity-fed into a 32 L (38L x 28W x 30H cm) experimental tank at 1.5 L.min^-1^.

Elevated-CO_2_ seawater was achieved by dosing the header tank with 100% CO_2_ to a set pH_NBS_ using a pH-controller (pH computer, Aqua Medic, Germany), following standard techniques [18]. A needle valve was used to regulate the flow of CO_2_ into the powerhead intake to ensure a slow, steady stream of fine CO_2_ gas bubbles during dosing. This slow dosing and rapid mixing in the header tank ensured that the experimental tank received a steady supply of well-mixed water.

The CO_2_ dosing system was set at a series of different pH_NBS_ levels throughout the experiment. A range of seawater pH_NBS_ values (8.2 to 7.6) were used, corresponding to ambient and elevated *p*CO_2_ of <400 to >1400 μatm, and measurements of seawater chemistry were taken in the experimental tank simultaneously using the four methods described below. Briefly, air equilibrated with seawater from the experimental tank passed across a NDIR CO_2_ gas analyser until CO_2_ levels had stabilised (*c*. 1 hr). Once CO_2_ readings were stable, data on CO_2_, pH_NBS_ and temperature were recorded. Water samples were taken for immediate spectrophotometric analysis of pH_T_, and preserved for later analysis of C_T_ and A_T_. Full details are described below.

### Quantification of carbonate chemistry parameters

#### 1) Determination of seawater dissolved inorganic carbon (C_T_) and total alkalinity (A_T_)

Water samples taken from the experimental tank at the time of measurement were immediately poisoned with a saturated solution of mercuric chloride (at 0.05% of the sample volume) and later analysed *in vitro* for C_T_ and A_T_ at the Australian Institute of Marine Sciences (AIMS) on a Versatile INstrument for the Determination of Total inorganic carbon and titration Alkalinity (VINDTA 3C, MARIANDA, Kiel, Germany). The VINDTA 3C was configured with a UIC Coulometer (model 5015) and UIC Anode Reagent and Cathode Reagent (UIC Inc., Joliet, Illinois, U.S.A.) for C_T_ analysis and a Metrohm Titrino titrator (model 702, Metrohm, Switzerland) with 0.1M HCl (fortified with NaCl to the ionic strength of seawater) added in 150 μl steps for A_T_ analysis, calculated by Gran titration. The VINDTA was calibrated with certified reference material (CRM) consisting of sterilized natural seawater of known C_T_ and A_T_ preserved with mercuric chloride (Prof. A.G. Dickson, Scripps Institution of Oceanography, U.S.A., batch number 126, one-point calibration). CRMs and samples were water-jacketed at 24°C and sample results were adjusted for salinity of the sample compared with the CRM. Since the VINDTA samples a fixed volume and the CRM is certified in mass units (μmol.kg^-1^), a small adjustment for the difference in the salinity of the sample compared with the salinity of the CRM at 24°C was required. Consequently, the raw VINDTA output of C_T_ and A_T_ was multiplied by seawater density at the CRM salinity, and divided by seawater density at the sample salinity. This adjustment reduced the raw VINDTA output of C_T_ and A_T_ by approximately 2–3 μmol.kg^-1^ to produce the final C_T_ and A_T_ measures.

A_T_ data were used as the second parameter in carbonate chemistry calculations for each of the four methods. Carbonate chemistry parameters derived from C_T_ and A_T_ were used to compare carbonate chemistry parameters determined from the other three methods. Reported measurement uncertainty for C_T_ and A_T_ using state-of-the-art methods with reference materials is 2–3 and 2–3 μmol.kg^-1^, respectively [17].

#### 2) Spectrophotometric determination of seawater pH_T_

Seawater pH on the total hydrogen ion concentration scale (total scale, pH_T_) was measured *in vitro* using a spectrophotometer following standard operating procedures (SOP 6b; [19]). The SOP was adapted for field use by using a compact, single-beam spectrophotometer (Spectronic 20 Genesys) and a spectrophotometric cell made of optical glass with a 10 mm path-length. This more compact system allowed transportation to the field site. Seawater pH determination was performed using the indicator dye meta/*m*-cresol purple (mCP) (*m*-cresol purple sodium salt 99%, non-purified, Acros Organic).

A seawater sample for spectrophotometric determination of pH_T_ was taken from the experimental tank underwater with no headspace, at the same time that all other seawater measurements and samples were taken. Absorbances of the cell + seawater were measured and recorded at the non-absorbing wavelength (730 nm) and at the dye absorption maxima (578 and 434 nm) as per SOP 6b [19]. Temperature of the sample during measurements was maintained to within ±0.1°C of 25.0°C and confirmed with a temperature probe (C26, Comark, Norwich, U.K.) before and after each spectrophotometric measurement. A highly accurate thermometer (Traceable^®^ Digital Thermometer 4000, Control Company, Texas, U.S.A.) was used to confirm the temperature probe reading was correct to within 0.1°C. During measurement, temperature was maintained within ≤0.1°C and any change in the non-absorbing wavelength at 730 nm was maintained within ≤0.001. These additional controls were employed to ensure maximum measurement quality at the field site. Additionally, spectrophotometer accuracy and stability were confirmed by replicate analysis of certified reference material (CRM) consisting of Tris buffer in synthetic seawater (Prof. A.G. Dickson, Scripps Institution of Oceanography, U.S.A., batch number 26, one-point calibration). Reported measurement uncertainty for pH using techniques with reference materials, other than state-of-the-art methods, is 0.01–0.03 pH units [17].

#### 3) Electrode measurement of seawater pH_NBS_

Seawater pH on the US National Bureau of Standards (NBS, an organisation now known as The National Institute of Standards and Technology) scale (pH_NBS_) was determined *in situ* with a portable, hand-held pH meter (SevenGo Pro pH/Ion, Mettler Toledo) and glass electrode (InLab^®^413 S8, Mettler Toledo) calibrated with certified reference materials (CRMs) for NBS consisting of pH_NBS_ 4 and 7 buffer solutions (Mettler Toledo, two-point calibration). Reported measurement uncertainty for pH using techniques with reference materials, other than state-of-the-art methods, is 0.01–0.03 pH units [17].

#### 4) Measurement of seawater CO_2_ with a non-dispersive infrared (NDIR) gas analyser

**Fig 1:**
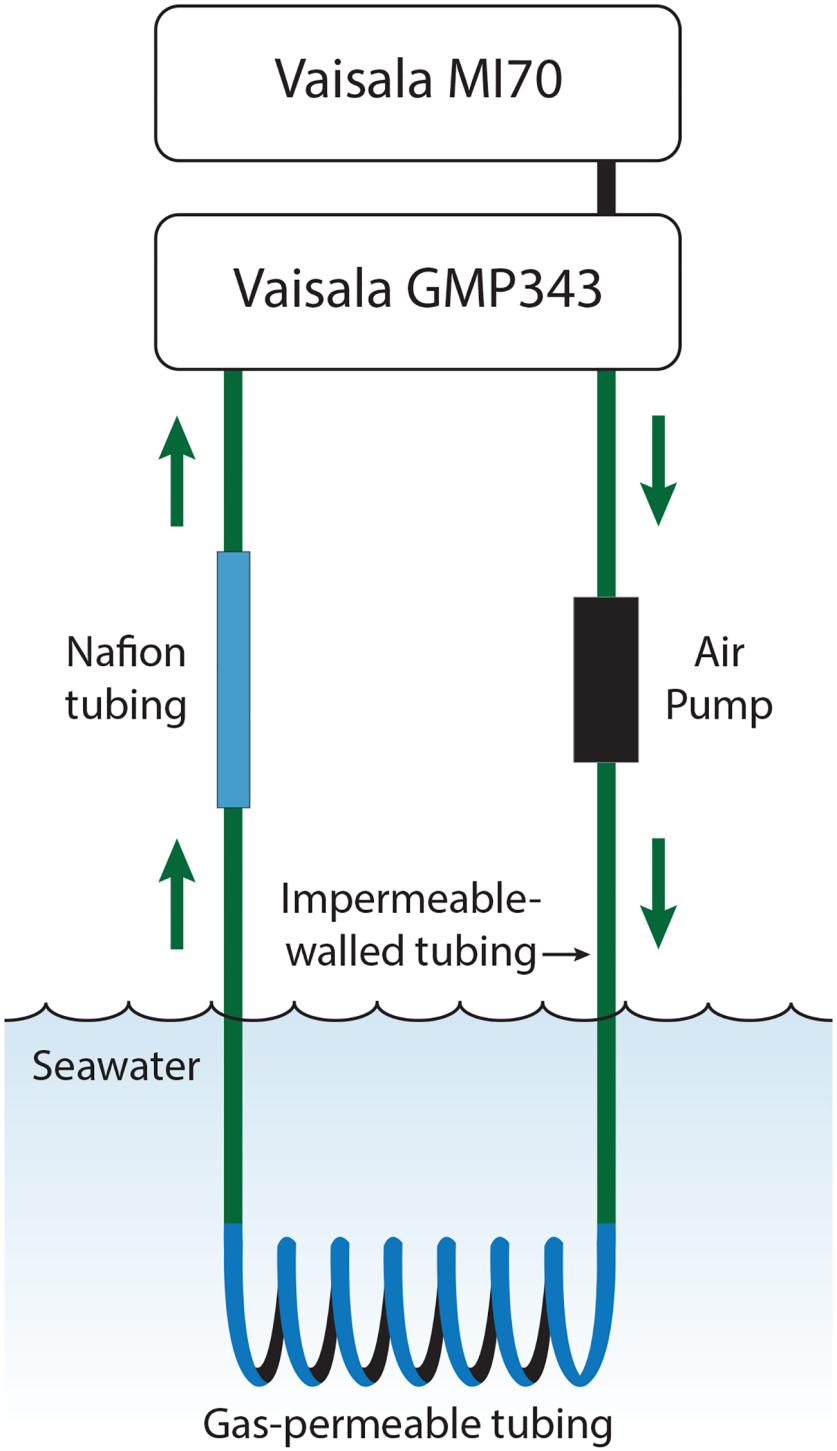
Diagram of the portable NDIR CO_2_ equilibrator. The portable CO_2_ equilibrator consists of a commercially available non-dispersive infrared (NDIR) CO_2_ gas analyser and data logger display interface, in-line air pump, impermeable-walled tubing and a section of gas-permeable membrane that is submerged in water. Air is pumped in a closed loop around the system and equilibrates with CO_2_ in seawater.

Seawater CO_2_ was measured *in situ* with a portable CO_2_ equilibrator with a high-resolution non-dispersive infrared (NDIR) gas analyser. This method for the direct measurement of CO_2_ in seawater using a NDIR sensor, described in more detail below, is taken from Hari et al. [20]; see also Munday et al. [21]. The portable CO_2_ equilibrator consisted of a NDIR CO_2_ sensor (CARBOCAP^®^ Carbon Dioxide Probe GMP-343, Vaisala, Helsinki, Finland, calibrated by Vaisala using certified reference materials (CRMs, six-point calibration) two months prior to the study) and data logger (Measurement Indicator MI70, Vaisala, Helsinki, Finland), air pump, gas-tight tubing, gas-permeable tubing and dehumidifying tubing (Fig 1). The NDIR CO_2_ sensor range was pre-programmed from 0 to 5000 ppm CO_2_ and the environmental settings on the data logger were set to 80.0% relative humidity, 1010.0 hPa ambient pressure and 21.0% oxygen. CO_2_ data from the sensor were compared directly with estimated *p*CO_2_ from the three other methods. The CO_2_ sensor was connected to the data logger that also served as a data display and interface, allowing visualisation of real-time as well as recorded CO_2_ data. Both the CO_2_ sensor and display interface were enclosed in a water-resistant plastic container.

A gas-permeable membrane (medical silicone tubing ID 3.0 mm, OD 3.8 mm, length 12.2 m) was coiled around rigid plastic mesh and connected to the CO_2_ sensor via gas-impermeable tubing (length 2.1 m, ID 4 mm, OD 6 mm) in a closed loop. A 60 cm length of Nafion^®^ membrane tubing (ID 2.18 mm, OD 2.74 mm, ME-110-24BB, Perma Pure LLC, Lakewood, NJ, U.S.A.), selectively permeable to only water vapour, in-line between the gas-permeable membrane and the CO_2_ sensor removed moisture from the air in the closed loop, if the humidity was greater than ambient, before it reached the sensor. A small 12 V AC closed circuit diaphragm pump (Rietschle Thomas miniature rotary vane pump, model G 01-K) was used to circulate air around the closed-loop system at a flow rate of 1.1 L.min^-1^. Once the circuit was closed, the gas-permeable membrane was submerged in seawater in the experimental tank and the in-line air pump turned on. This allowed the air inside the closed loop to equilibrate with seawater CO_2_ over time (S1 Fig). Including the water-resistant housing, the total system weighed 1.4 kg. Adding the 1.4 kg 12 V AC power transformer gave a combined total weight of 2.8 kg, and compact packed size of 26L x 23W x 17H cm.

CO_2_ data from the sensor were generated every 2 seconds and mean values recorded every minute by the data logger. Values were logged until CO_2_ readings stabilised. The graph plot on the MI70 was used to visualise data to ensure an equilibrium state was reached (stable plateau of the graph, S1 Fig). The seawater CO_2_ value was recorded when the system was at equilibrium. Data files stored on the data logger were downloaded using the software MI70 Link (version 1.06, Vaisala, 2002). Reported accuracy of the GMP-343 sensor configuration used is ±13 ppm at 400 ppm CO_2_, ±25 ppm at 1000 ppm CO_2_, and ±33 ppm CO_2_ at 1400 ppm CO_2_.

### Carbonate chemistry calculations

Carbonate chemistry parameters were calculated in CO2SYS [22] using the constants K1, K2 from Mehrbach *et al*. 1973 refit by Dickson & Millero 1987, and Dickson for *K*(HSO_4_^-^). The pH_NBS_ scale was used for calculations in CO2SYS using pH_NBS_ electrode data and the pH_T_ scale was used for calculations using data from spectrophotometric pH_T_. For each of the four methods, raw data are presented, and have not been adjusted for any offset compared with expected values from certified reference materials (CRMs). Seawater temperature was measured with a temperature probe (C26, Comark, Norwich, U.K.). Temperature during the experiment in this open system was 26.9 ± 0.7°C (mean ± s.d.). Salinity data were obtained from moorings around Lizard Island, which form part of the Australian National Mooring Network Integrated Marine Observing System (IMOS) operated by the Australian Institute of Marine Science [23]. During the study, salinity was 35.4 ± 0.0 (mean ± s.d.) and A_T_ was 2291.8 ± 5.6 μmol.kg^-1^ (mean ± s.d.). Levels of total P and Si in seawater were below detection limits (total phosphorus <3.2 μmol.kg^-1^ SW as P, silica <8.1 μmol.kg^-1^ SW), and thus set to 0 for calculations in CO2SYS.

### Data analysis

Estimates of *p*CO_2_ were compared among methods using generalised linear models (GLM) with the statistical software R [24]. A Gaussian distribution was used to assess the relationship between *p*CO_2_ estimates derived from the three different methods against those derived from C_T_ and A_T_, while the log-link function and quasipoisson distribution were used to compare estimated aragonite saturation state against the estimated *p*CO_2_ values. Estimated measurement uncertainties were calculated for each method by determining the relative difference in each carbonate chemistry parameter from values derived from C_T_ and A_T_ as a reference. The root mean square error (RMSE) (= root mean square deviation, RMSD) [25] was then determined for each method for *p*CO_2_, the saturation state of seawater with respect to aragonite (Ω_ar_) and [H^+^] (Table 2). Absolute differences were also calculated by taking the mean of the deviations (as positive numbers) for each measurement and are reported in the text for *p*CO_2_, Ω_ar_ and pH.

**Table 2:** Estimated measurement uncertainties associated with each method determined from C_T_ and A_T_-derived reference values.

Parameter	Spectrophotometric pH_T_ and A_T_ uncertainty (%)	Electrode pH_NBS_ and A_T_ uncertainty (%)	NDIR CO_2_ equilibrator uncertainty (%)
***p*CO**_**2**_	4.6	3.6	3.5
**Ω**_**ar**_	3.3	2.7	2.7
**[H**^**+**^**]**	3.9	3.1	3.0

## Results and discussion

**Fig 2:**
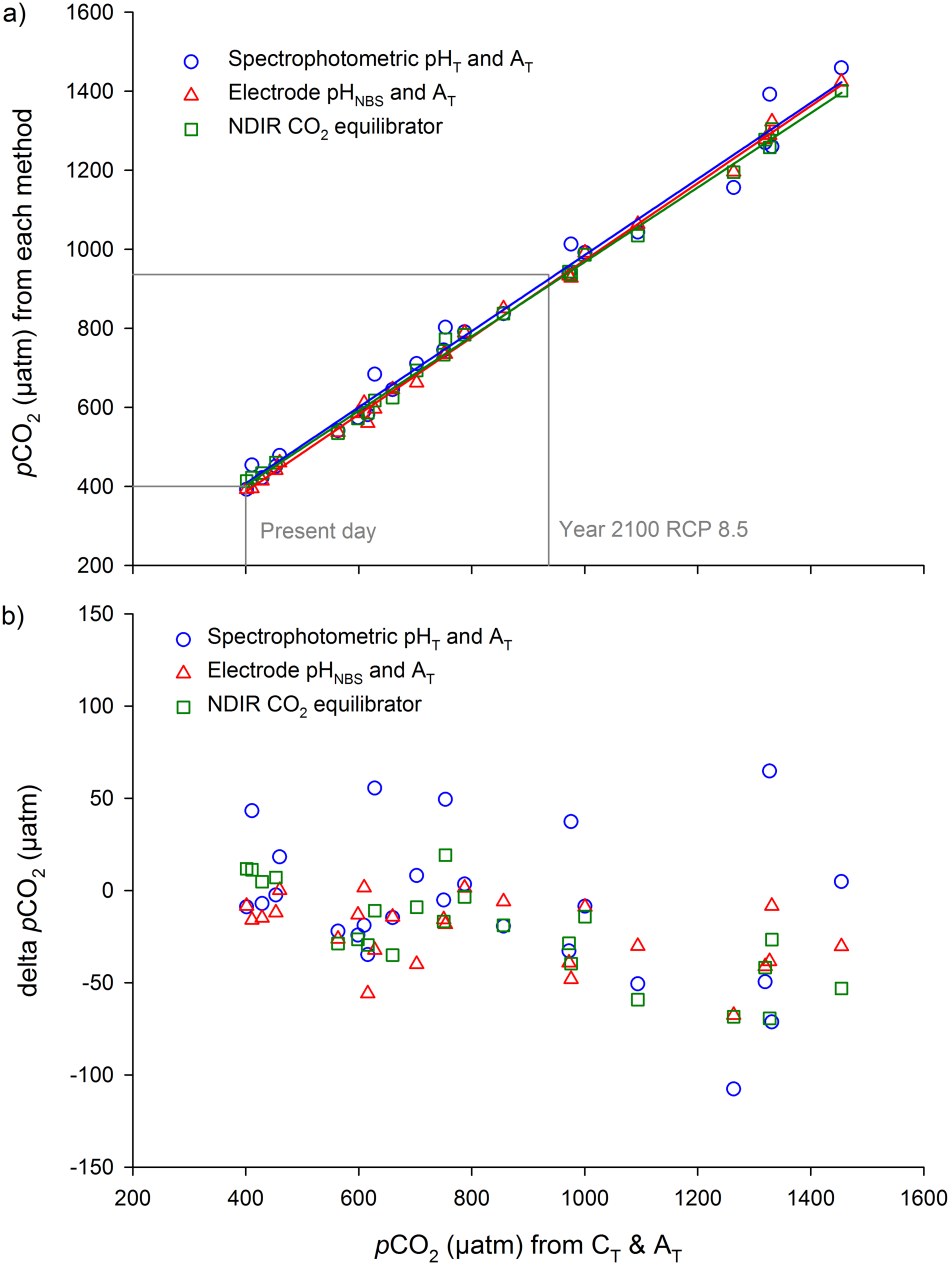
Seawater *p*CO_2_ calculated from C_T_ and A_T_, compared with three other methods: 1) spectrophotometric pH_T_ and A_T_ (n = 25), 2) electrode pH_NBS_ and A_T_ (n = 25), and 3) the direct measurement of seawater CO_2_ with a NDIR CO_2_ equilibrator (n = 23); a) for *p*CO_2_ data and b) for the difference in *p*CO_2_ compared to *p*CO_2_ derived from C_T_ and A_T_ (delta *p*CO_2_).

All four methods: 1) C_T_, 2) spectrophotometric pH_T_, 3) electrode pH_NBS_, and 4) the CO_2_ equilibrator, were compared across the *p*CO_2_ range tested in this study: 370 to 1460 μatm (Fig 2). Estimated measurement uncertainties for *p*CO_2_ from spectrophotometric pH_T_, electrode pH_NBS_ and the CO_2_ equilibrator were ≤4.6% (Table 2). Overall, there was no effect of method on *p*CO_2_ data when compared with *p*CO_2_ data derived from C_T_ and A_T_ (GLM analysis after exclusion of the non-significant interaction terms between *p*CO_2_ and method: F_2,69_ = 2.60, p = 0.082, Fig 2).

**Fig 3:**
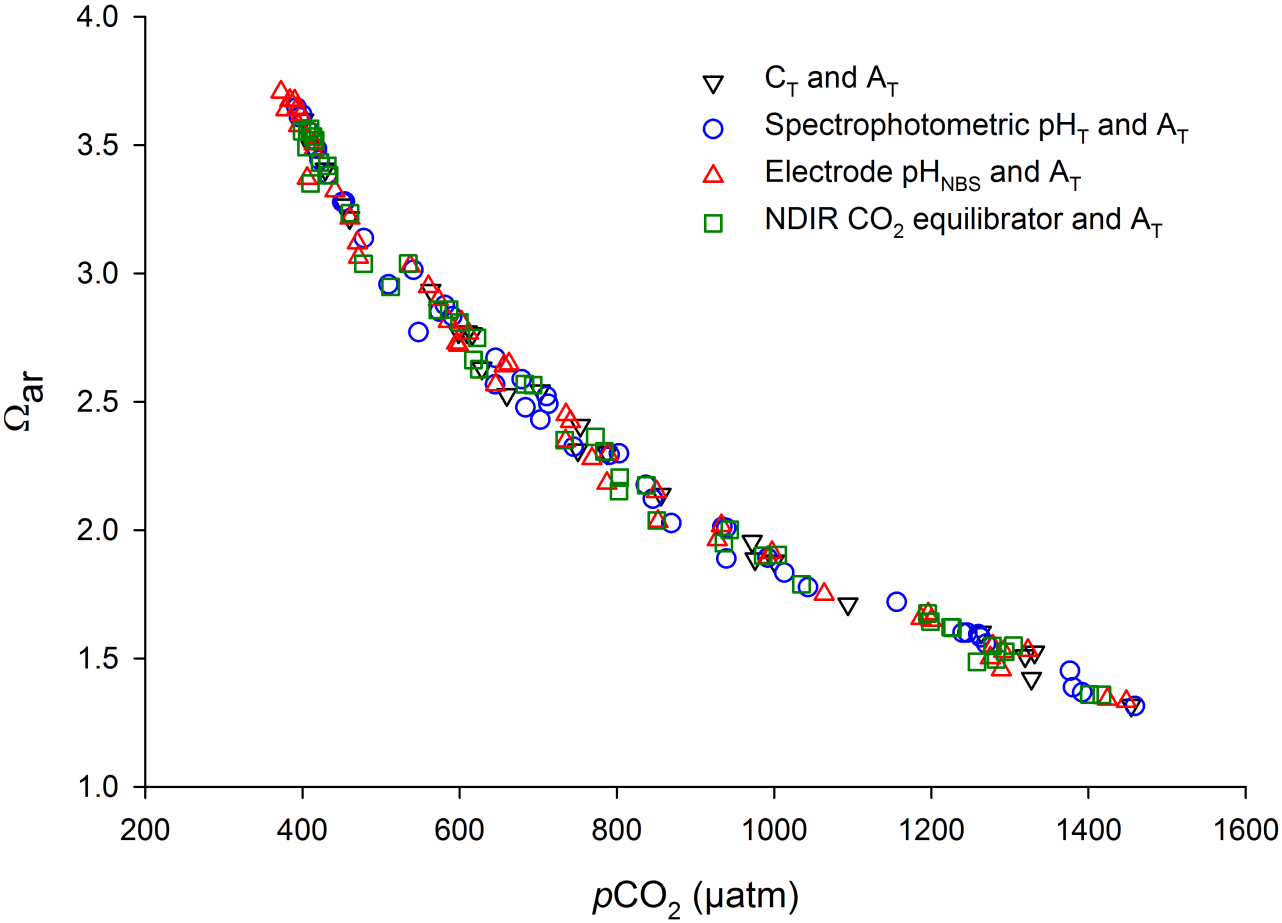
Relationship of seawater *p*CO_2_ and aragonite saturation state (Ω_ar_) determined by four different methods: 1) C_T_ and A_T_ (n = 25), 2) spectrophotometric pH_T_ and A_T_ (n = 45), 3) electrode pH_NBS_ and A_T_ (n = 49), and 4) the direct measurement of seawater CO_2_ with a NDIR CO_2_ equilibrator (n = 46).

A comparison of the four methods (C_T_, spectrophotometric pH_T_, electrode pH_NBS_ and CO_2_ equilibrator), showed there was no difference in their estimates of *p*CO_2_ and aragonite saturation (GLM analysis after exclusion of the non-significant interaction terms between *p*CO_2_ and method: F_3,160_ = 0.148, p = 0.931, Fig 3). Each of the four methods is discussed in more detail below.

### 1) Determination of seawater dissolved inorganic carbon (C_T_) and total alkalinity (A_T_)

The combination of C_T_ and A_T_ is currently the preferred method for the characterisation of open ocean carbonate chemistry, and certified reference materials (CRMs) (sterilized natural seawater) for C_T_ and A_T_ are readily available [17] to ensure the accuracy and reliability of C_T_ and A_T_ determination. In this study, measurement standard deviations of C_T_ and A_T_ were within 3 and 2 μmol.kg^-1^ of CRMs, respectively, determined from repeat analysis of CRMs, run in conjunction with study samples. The sample processing time (approx. 23 min per sample) for C_T_ and A_T_ on the VINDTA allowed 7–8 samples to be processed per standard working day after appropriate machine warm-up (*c*. 2 hr) and control with CRMs, or 16–20 samples during an extended 11–12 hr shift.

In addition to the ease of availability of CRMs, the advantage of using C_T_ as a carbonate chemistry parameter is that water samples can be poisoned and analysed later at a convenient time. The disadvantages of C_T_, however, are that 1) access to equipment to measure C_T_ (such as a VINDTA) can be limited and may be costly, 2) the often long time lag to obtain results with no immediate data for field or lab CO_2_ perturbation experiments, and 3) the requirement to take and store many water samples. Additionally, water samples collected for C_T_ measurement must be air-tight as C_T_ values are affected by gas exchange. The disadvantages of preserved water samples include the fact they are heavy and freight can therefore be costly, that hazardous chemicals (mercuric chloride) are required to fix the samples, and that the shipment of seawater as corrosive and dangerous goods is controlled nationally and restricted internationally through customs. Hazardous chemicals also require specialist facilities for use (e.g. appropriate protective equipment) and proper disposal.

The advantages and disadvantages of A_T_ are the same as those for C_T_, except that access to titration equipment, such as an automatic titrator or manual titration equipment, is more readily available and less costly, and A_T_ measurement is not prone to gas exchange. As such, A_T_ is routinely used as a second parameter in combination with other techniques.

### 2) Spectrophotometric determination of seawater pH_T_

Seawater pH measured with a spectrophotometer using a procedure adapted from SOP 6b [19] for field station use produced values within a range of -0.0048 to 0.0087 (0.0012 ± 0.0045 mean ± s.d.) pH_T_ units of certified Tris buffer in synthetic seawater. An accuracy within 0.01 pH_T_ units of certified Tris buffer was achieved with the field system set-up used here, and replicate measures of the same seawater sample were within 0.005 pH_T_ units.

Seawater chemistry calculated with spectrophotometric pH_T_ and A_T_ produced *p*CO_2_ estimates close to those calculated from C_T_ and A_T_, with an average difference of 30.5 ± 26.1 μatm (mean ± s.d.). The estimated measurement uncertainty of *p*CO_2_ using the spectrophotometric pH_T_ and A_T_ technique was 4.6% (Table 2). Spectrophotometric pH_T_ values were on average within 0.014 ± 0.010 (mean ± s.d.) of pH_T_ values calculated from C_T_ and A_T_, and Ω_ar_ calculated from pH_T_ and A_T_ was on average within 0.06 ± 0.05 of Ω_ar_ values calculated from C_T_ and A_T_.

The advantage of the spectrophotometric pH_T_ method is that it produces pH values on the total scale (pH_T_). Measurement of pH on the total scale is preferred [17] given the ionic strength of seawater. However, the disadvantages are that certified reference materials (CRMs) for spectrophotometric pH_T_ (certified Tris buffer) are often limited [17], spectrophotometers may need custom modifications for seawater pH_T_ measurement and are unlikely to be available ‘off the shelf’ (SOP 6b; [19]), and dye impurities can affect measurement accuracy [26]. Other disadvantages are that spectrophometers can be large and bulky compared with pH electrodes and portable NDIR CO_2_ sensors, and traditional spectrophotometers may not be suitable for transport to field stations. Smaller spectrophotometers have, however, recently become available and may be better suited to field use than traditional spectrophotometers.

Measurement of spectrophotometric pH_T_ is an *in vitro* technique and requires more equipment and more time per sample than electrode pH measures. When working with seawater at temperatures >25°C, such as in the tropics, samples must be first cooled to 25°C. Consequently a standard heated water bath is not suitable, and a chiller bath or chilled room is required. We found achieving temperature precision (±0.1°C) whilst chilling water samples to the specific temperature required was time consuming in a field setting. Temperature adjustment (cooling) of the sample to laboratory temperature (25°C) required about 15–30 min. Sampling processing time was around 2–3 min per sample; however, if sample temperature or absorbance at the non-absorbing wavelength changed, then the sample was re-run until the quality control criteria were met. Consequently, a custom-manufactured chiller unit with precision temperature control could be useful for spectrophotometric pH_T_ measurement for tropical ocean acidification experiments. Variation in carbonate chemistry data from spectrophotometric pH_T_ was likely due to the challenges of maintaining constant temperature (lower then ambient in the tropics) during sample analysis, even in a temperature controlled room. A more recent study describes a formula to use *m*-cresol purple over a range of temperatures [26] which may circumvent the requirement to measure the samples at 25.0°C.

### 3) Electrode measurement of seawater pH_NBS_

Electrode pH_NBS_ measurement produced *p*CO_2_ estimates with an average difference of 23.5 ± 18.1 μatm (mean ± s.d.) compared with *p*CO_2_ estimates derived from C_T_ and A_T_. The estimated measurement uncertainty of *p*CO_2_ using the electrode pH_NBS_ and A_T_ technique was 3.6% (Table 2). Electrode pH_NBS_ values were on average within 0.011 ± 0.008 (mean ± s.d.) of pH_NBS_ values calculated from C_T_ and A_T_, and Ω_ar_ calculated from pH_NBS_ and A_T_ was on average within 0.05 ± 0.04 of Ω_ar_ values calculated from C_T_ and A_T_.

There are some advantages of electrode pH_NBS_ measurement. Electrodes produced the most rapid measurement of seawater chemistry of all techniques assessed in this study, stabilising initially in a few minutes or less, and then typically in one minute or less for subsequent measures. Measurements can be taken over a range of seawater temperatures (although in much cooler waters, electrodes can take longer to stabilise), and 2 or 3 point (or more) calibrations are possible using readily available reference materials. Thus using an electrode to measure pH can allow the measurement of many tanks (e.g. 50+) per day, which can be useful for large ecological experiments with many replicates and field-based studies. With careful electrode calibration with certified reference materials (CRMs) and further cross-checks of electrode pH_NBS_ measures against pH_NBS_ calculated from NDIR CO_2_ in combination with approximate expected or actual A_T_, we found that it is possible to achieve pH accuracy comparable to estimated measurement uncertainties reported from non-state-of-the-art techniques that use reference materials (0.01–0.03 pH units) [17]. The benefit of recording immediate carbonate chemistry data for multiple tanks, and thus enhanced tank data resolution, is significant because any tank differences can be detected rapidly during the experiment and appropriate action taken during the experiment or in the analyses.

The disadvantages of pH_NBS_ electrodes is that the uncertainty in measuring can be up to 0.05 pH_NBS_ units for seawater measurements [17]. However, with careful use our results indicate that improved accuracy within ≤0.02 pH_NBS_ units can be achieved. In general, and to achieve the greatest measurement certainty, we recommend electrode pH_NBS_ measurements are validated by cross-checking data with another method, such as one of the three other methods evaluated here, to ensure accurate results. This is important because undetected, the potential uncertainty (of up to 0.05 pH units) [18] from pH_NBS_ electrodes may create uncertainty in estimated *p*CO_2_ of around 50–150 μatm over the 375–1250 μatm *p*CO_2_ range often used in biological ocean acidification studies.

### 4) Measurement of seawater CO_2_ with a non-dispersive infrared (NDIR) gas analyser

The NDIR CO_2_ equilibrator system gave very similar *p*CO_2_ estimates to those derived from seawater chemistry using C_T_ and A_T_, with an average difference in CO_2_ values of 27.6 ± 19.8 μatm (mean ± s.d.). The estimated measurement uncertainty of *p*CO_2_ using the CO_2_ equilibrator was 3.5% (Table 2). Ω_ar_ calculated from equilibrator CO_2_ values and A_T_ was on average within 0.05 ± 0.03 (mean ± s.d.) of Ω_ar_ values calculated from C_T_ and A_T_.

The ability to measure *p*CO_2_ directly in seawater is particularly beneficial, firstly because *p*CO_2_ is the key experimental target condition in many biological ocean acidification perturbation experiments, and secondly, because direct *p*CO_2_ measurement allows for appropriate *p*CO_2_ dosing in manipulation experiments when analysis of other carbonate chemistry parameters, such as A_T_, is not immediately available. Recording *p*CO_2_ directly could also save some of the time and cost required to process other seawater carbonate chemistry parameters (e.g. pH, A_T_ or C_T_, and the associated equipment) if *p*CO_2_ is the principal carbonate chemistry parameter of interest in a study. Improved confidence in seawater carbonate chemistry can be achieved if A_T_ is confirmed as a common unchanging value in experiments.

The CO_2_ equilibrator itself too has several advantages. It is simple, portable, relatively low cost and reasonably rugged. Conveniently, CO_2_ data are available in close to real-time. The closed-loop takes some time to equilibrate which makes the time lag to obtain results longer than electrode pH_NBS_, but on a par with spectrophotometric pH_T_. The time taken to reach equilibrium with a 12.2 m length of gas-permeable tubing was up to approximately 1 hour for each measurement (S1 Fig). Faster equilibrium times can be achieved if the starting CO_2_ level is closer to the final CO_2_, or if a longer length of gas-permeable tubing and/or shorter length of impermeable tubing or smaller diameter tube was used to reduce total system:permeable tubing air volume ratio. Potentially separate coils could be used and connected in turn to one NDIR CO_2_ sensor close to stabilisation time to accelerate the process of obtaining measurements from multiple tanks.

The CO_2_ equilibrator described here can be used in small bodies of water *c*. 10–20 litres in volume, and smaller versions can be easily made for even smaller water bodies (<5–10 litres). Alternatively, the equilibrator can be modified to use a ‘shower head’ device to spray seawater into a closed loop of air for use with small volumes of water and to reduce equilibration time. This shower head method is, however, more bulky in size than the membrane coil and consequently less portable for field use. Notably, the CO_2_ equilibrator tested here provides a portable system that is light-weight and compact suitable for measurement in field laboratory situations, and is not intended to be compared to underway CO_2_ measuring systems such as that described by Bandstra et al. [27].

In summary, the CO_2_ equilibrator tested here is a simple, small, lightweight, relatively low cost device that provides a method for the direct measurement of CO_2_ in water and is suitable for laboratory and field-based experimental studies. It is robust enough for use at field locations where pH may be the only other parameter of seawater carbonate chemistry that is immediately measurable. The CO_2_ equilibrator can thus provide cost-effective, near real-time estimates of *in-situ* seawater *p*CO_2_ for biological experiments, providing a major advantage to biological perturbation experiments where achieving a desired *p*CO_2_ is key.

### Evaluation

In combination with A_T_ as the second carbonate chemistry parameter, all four methods produced very similar *p*CO_2_ estimates, and the three field methods 1) spectrophotometric pH_T_, 2) electrode pH_NBS_, and 3) NDIR CO_2_ equilibrator, performed comparably to C_T_ with careful use. In this study, electrode pH_NBS_ and the CO_2_ equilibrator gave consistently close results to C_T_-derived *p*CO_2_ values, and had the smallest ranges. Spectrophotometric pH_T_ produced *p*CO_2_ values that were on average slightly further from C_T_-derived *p*CO_2_, compared with electrode pH_NBS_ and the CO_2_ equilibrator. All methods calculated Ω_ar_ within ≤0.06, which is within the recommended <0.2 units [28].

When choosing a technique to use, consideration should be given to the required precision and accuracy, and the number and frequency of measures required. For example, rapid methods such as electrode pH_NBS_ can provide the scope to measure many tanks requiring daily or more frequent assessment, whereas lower frequency techniques including methods that require water samples may be more suitable for experiments with fewer replicates. Due consideration should be given to the potential uncertainty inherent in all techniques, which can be larger for some methods, such as pH_NBS_, without careful use. Consequently, the use of reference materials, and cross-validation wherever possible, is strongly recommended for all methods used.

Although sample measurement time is not necessarily long for C_T_ and A_T_ (i.e. 10–25 min once the system is calibrated and running), the limited availability of instruments to measure these at field sites often means such water samples are batch processed at a later time, often after the end of the experiment. The time lag to obtain results therefore becomes an important consideration. Spectrophotometric pH_T_, electrode pH_NBS_ and the CO_2_ equilibrator provide data in real-time or near real-time (Table 1).

Other considerations in method choice include the availability of equipment and reference materials, and cost. For example, some techniques require sophisticated equipment, such as a thermostated spectrophotometer cell (e.g. SOP 6b; [19]) or VINDTA, which may not be available ‘off-the-shelf’ and require further custom manufacturing. Certified reference materials for techniques such as spectrophotometric pH_T_ may also be difficult to acquire [17]; certified Tris buffers (from Prof. A.G. Dickson, Scripps Institution of Oceanography, U.S.A.), for example, are often in short supply. One option may be to collaborate with research groups who have access to the required equipment to ensure that carbonate chemistry quality is not compromised.

## Conclusions and recommendations

Our results indicate that the portable CO_2_ equilibrator used in conjunction with one of the other methods described here (C_T_, spectrophotometric pH_T_, or electrode pH_NBS_) provides a suitable combination for estimating and maintaining *p*CO_2_ levels in biological ocean acidification experiments. The other three methods (C_T_, spectrophotometric pH_T_, or electrode pH_NBS_) all require a second carbonate chemistry parameter in order to determine *p*CO_2_, and all four methods require a second carbonate chemistry parameter to calculate other parameters of the seawater carbonate chemistry system. A_T_ is well suited for this purpose, and the measurement or calculation of A_T_ is also useful to characterise the seawater used in the experiment. For perturbation experiments that manipulate C_T_ (such as CO_2_ injection), where A_T_ remains constant, limited numbers of A_T_ samples can be taken and analysed later (e.g. after the experiment). During the experiment, a NDIR sensor coupled with a CO_2_ equilibrator can be used to ensure seawater *p*CO_2_ in manipulation experiments is correct.

For all techniques, we recommend the used of certified reference materials (CRMs) to ensure high quality control for seawater carbonate chemistry and we recommend cross-checking measurements with another technique to further ensure quality control wherever possible. For example, cross-checking electrode pH_NBS_ measures against pH_NBS_ calculated from NDIR CO_2_ in combination with expected A_T_ can reduce uncertainty associated with electrode pH_NBS_ measures whilst still allowing for high frequency sampling, such as in studies with high tank replication.

Importantly, we are not advocating the replacement of established methods to measure open ocean chemistry and constrain the ocean CO_2_ system for real-time measures of ocean change, nor for the measurement of small changes in seawater *p*CO_2_. However, for biological perturbation experiments measuring differences of over 100 μatm *p*CO_2_ among treatments, we find the four methods described here can be adequate and with careful use they can all produce similar results. Although methods such as the portable CO_2_ equilibrator and pH_NBS_ electrodes do not replace standard methods, such as C_T_ and spectrophotometric pH_T_, for high-accuracy quantification of carbonate parameters in seawater; they can, provide a cost-effective means to determine *p*CO_2_ in large ecological experiments investigating the effects of ocean acidification on marine organisms providing options for greater tank and temporal resolution.

In summary, we show that all four combinations of methods tested here 1) C_T_ and A_T_, 2) spectrophotometric pH_T_ and A_T_, 3) electrode pH_NBS_ and A_T_, and 4) the NDIR CO_2_ equilibrator, can achieve *p*CO_2_ values accurate enough for biological ocean acidification manipulation experiments with careful use. In addition, we find the portable NDIR CO_2_ equilibrator tested provides a cost-effective system for near real-time measures of CO_2_. For all methods, we recommend the used of certified reference materials (CRMs) and cross-checking data with another method to ensure quality control in biological ocean acidification experiments.

## Supporting information

S1 Fig CO_2_ measurements recorded by the portable CO_2_ equilibrator over time from the start of a test period until equilibrium is reached (boxed area). Stabilisation time was 1 hour. This time period is a conservative estimate since equilibration time is shorter if the *p*CO_2_ difference between two samples is less.
(PDF)
