# Supplementary material for: Quantifying pCO2 in biological ocean acidification experiments: A comparison of four methods
Source: PLoS One. 2017 Sep 28;12(9):e0185469. doi: 10.1371/journal.pone.0185469 (PMC5619781; doi:10.1371/journal.pone.0185469)
Supplement: S1 Fig — Stabilisation time was 1 hour. This time period is a conservative estimate since equilibration time is shorter if the pCO2 difference between two samples is less. (PDF) [file pone.0185469.s001.pdf]

Quantifying pCO_2 in biological ocean acidification experiments: a comparison of four methods

Sue-Ann Watson, Katharina E. Fabricius and Philip L. Munday

Supplementary figure

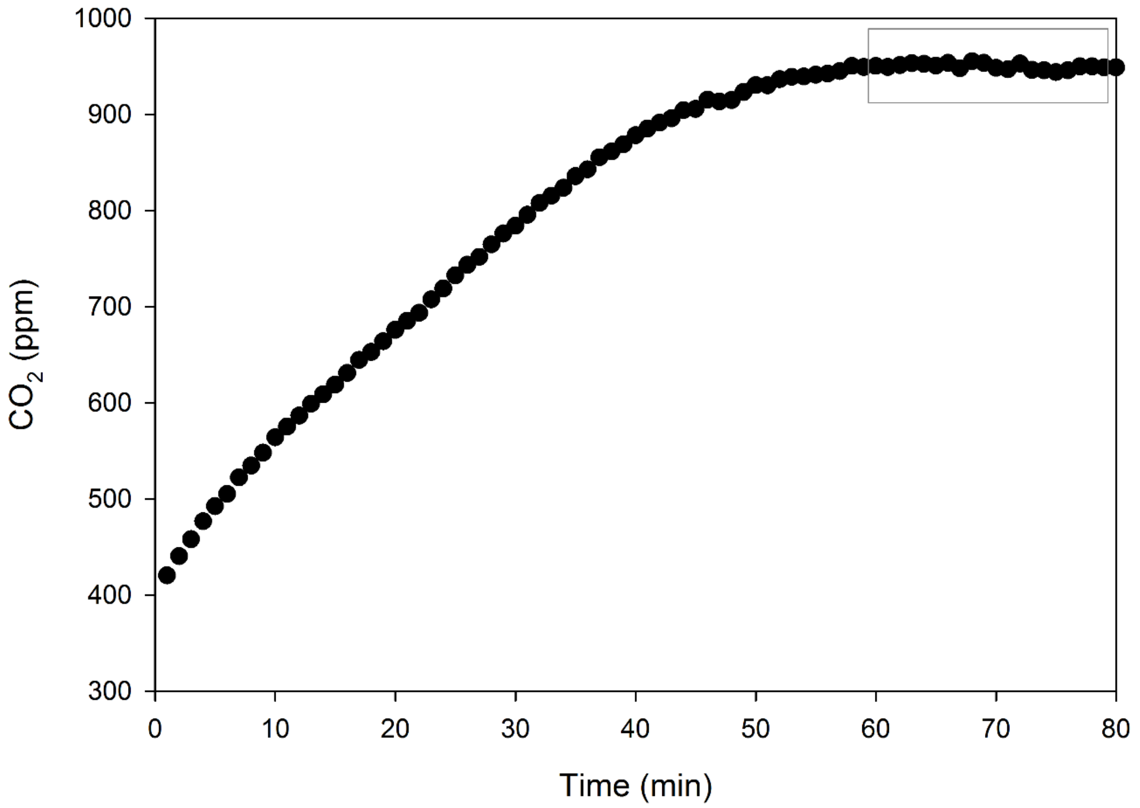

S1 Fig. CO_2 measurements recorded by the portable CO_2 equilibrator over time from the start of a test period until equilibrium is reached (boxed area). Stabilisation time was 1 hour. This time period is a conservative estimate since equilibration time is shorter if the pCO_2 difference between two samples is less.
